# The Transfection of BDNF to Dopamine Neurons Potentiates the Effect of Dopamine D3 Receptor Agonist Recovering the Striatal Innervation, Dendritic Spines and Motor Behavior in an Aged Rat Model of Parkinson’s Disease

**DOI:** 10.1371/journal.pone.0117391

**Published:** 2015-02-18

**Authors:** Luis F. Razgado-Hernandez, Armando J. Espadas-Alvarez, Patricia Reyna-Velazquez, Arturo Sierra-Sanchez, Veronica Anaya-Martinez, Ismael Jimenez-Estrada, Michael J. Bannon, Daniel Martinez-Fong, Jorge Aceves-Ruiz

**Affiliations:** 1 Departamento de Fisiología, Biofísica y Neurociencias, Centro de Investigación y de Estudios Avanzados del Instituto Politécnico Nacional (CINVESTAV), México D.F., México; 2 Neuromorphology Lab, Facultad de Estudios Superiores Iztacala, Universidad Nacional Autonoma de México (UNAM), Tlalnepantla, Estado de México, México; 3 Department of Pharmacology, Wayne State University School of Medicine, Detroit, Michigan, United States of America; 4 Programa de Nanociencias y Nanotecnología, Centro de Investigación y de Estudios Avanzados del Instituto Politécnico Nacional (CINVESTAV), México D.F., México; Florey Institute of Neuroscience and Mental Health, The University of Melbourne, AUSTRALIA

## Abstract

The progressive degeneration of the dopamine neurons of the pars compacta of substantia nigra and the consequent loss of the dopamine innervation of the striatum leads to the impairment of motor behavior in Parkinson’s disease. Accordingly, an efficient therapy of the disease should protect and regenerate the dopamine neurons of the substantia nigra and the dopamine innervation of the striatum. Nigral neurons express Brain Derived Neurotropic Factor (BDNF) and dopamine D3 receptors, both of which protect the dopamine neurons. The chronic activation of dopamine D3 receptors by their agonists, in addition, restores, in part, the dopamine innervation of the striatum. Here we explored whether the over-expression of BDNF by dopamine neurons potentiates the effect of the activation of D3 receptors restoring nigrostriatal innervation. Twelve-month old Wistar rats were unilaterally injected with 6-hydroxydopamine into the striatum. Five months later, rats were treated with the D3 agonist 7-hydroxy-N,N-di-n-propy1-2-aminotetralin (7-OH-DPAT) administered i.p. during 4½ months via osmotic pumps and the BDNF gene transfection into nigral cells using the neurotensin-polyplex nanovector (a non-viral transfection) that selectively transfect the dopamine neurons via the high-affinity neurotensin receptor expressed by these neurons. Two months after the withdrawal of 7-OH-DPAT when rats were aged (24 months old), immunohistochemistry assays were made. The over-expression of BDNF in rats receiving the D3 agonist normalized gait and motor coordination; in addition, it eliminated the muscle rigidity produced by the loss of dopamine. The recovery of motor behavior was associated with the recovery of the nigral neurons, the dopamine innervation of the striatum and of the number of dendritic spines of the striatal neurons. Thus, the over-expression of BDNF in dopamine neurons associated with the chronic activation of the D3 receptors appears to be a promising strategy for restoring dopamine neurons in Parkinson’s disease.

## Introduction

Parkinson´s disease is a progressive neurodegenerative disorder characterized clinically by bradykinesia, reduced motor coordination, muscle rigidity, and gait dysfunction, among other alterations of motor behavior. The clinical decline reflects ongoing nigrostriatal dopaminergic degeneration [1,2]. Levodopa continues to be the best treatment for the disease, because it eliminates most of the motor symptoms; however, it does not prevent the progressive loss of dopamine neurons and produces, after 4–5 years of treatment, dyskinesia. The progressive neuronal degeneration ultimately results in severe motor, mental and functional disability. This suggests that a therapeutic strategy preventing neuronal death and promoting growth and regeneration would be a valuable approach to control the disease [3]. Several neurotrophic factors have been evaluated as potential neuro-protective agents [4–6]. The trophic effect of BDNF on dopamine neurons is well established [7,8]. BDNF is expressed by dopamine neurons of substantia nigra pars compacta where it plays a critical role in functions such as cell proliferation [9], cell survival [10], synaptic plasticity [11], dopamine release modulation [12,13], neuronal firing [14], striatal re-innervation [15], dopamine phenotype induction [8,16] and dopamine D3 receptor expression [17,18]. In addition, nigral dopamine neurons degenerate in the absence of BDNF, suggesting its involvement in the pathogenesis of Parkinson´s Disease [19,20]. The reduced expression of BDNF in nigral neurons in Parkinson´s disease patients and in rats with lesions of the nigrostriatal innervation also suggests its participation in the pathogenesis of the disease [19,21,22]. Its reduction in the disease is not only because of the loss of the dopamine neurons but also because the remaining neurons express less BDNF [19].

The activation of dopamine D3 receptors also has trophic effects. It increases the neurogenesis in the subventricular zone and neostriatum in the adult rat brain via rapidly amplifying progenitor cells [23,24]. The activation of D3 receptors stimulates mitogenesis [25–27] and increases the arborization of the dendrites of mesencephalic dopaminergic neurons [28]. The reduced expression of this receptor in Parkinson´s disease patients and in rats with lesions of the nigrostriatal innervation also suggests its participation in the pathogenesis of the disease [29,30]. In addition, the selective activation of dopamine D3 receptors regenerates the nigrostriatal pathway, thus improving some aspects of motor behavior [31].

BDNF mediates, in part, the trophic effect of the activation of dopamine D3 receptors [32], in turn, BDNF increases the number of D3 receptors [17,18]. In addition, dopamine increases the number of TH+ neurons isolated from the cerebral cortex of 14–15 day-old embryos, and BDNF markedly increases the enhancement in the number TH+ neurons generated by dopamine [8]; it is possible that this effect was mediated by D3 receptors because they first appear in embryos of about the same age [33]. There exists then the possibility that BDNF potentiates the trophic effect of the activation of dopamine D3 receptors in the adult brain. We explored here this possibility by examining whether BDNF potentiates the regeneration of nigrostriatal innervation brought about by the activation of D3 receptors [31]. Preliminary results of this work have been presented elsewhere [34]

## Materials and Methods

### Ethics Statement

All procedures were carried out in strict accordance with the current Mexican legislation, NOM-062-ZOO-1999 and NOM-087-ECOL-1995 (SAGARPA), based on the Guide for the Care and Use of Laboratory Animals, NRC. The CINVESTAV Institutional Animal Care and Use Committee approved our procedures for animal use (protocol #0121–03). All efforts were made to minimize animal suffering. Anesthetics (chloral hydrate anesthesia, 300 mg/kg, i.p.) and analgesics (ketoprofen 1%, 0.2 ml i.p.) were used when appropriate under the direction of a veterinarian to alleviate potential pain and distress and were given prior to euthanasia with the transcardial perfusion with 120 ml of 0.9% saline, followed by 300 ml of 4% paraformaldehyde.

### Experimental Protocol

**Fig 1:**
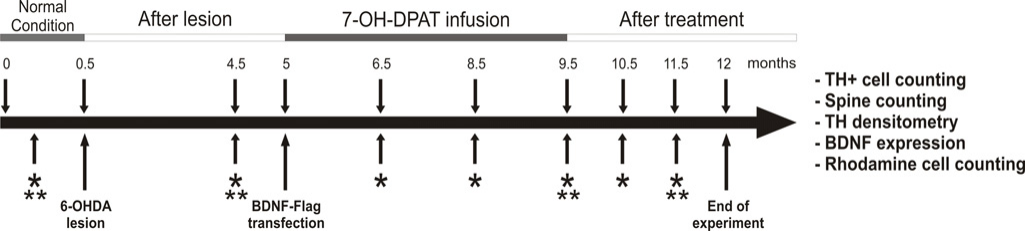
Illustration of the experimental design. Arrows with asterisk indicate an evaluation of the motor behavior, which included gait analysis, rotarod performance and ambulatory activity in the open field. Double asterisks indicate EMG recordings. Rats were 12 months old at the beginning of the experiment.

Male adult Wistar rats were housed individually when appropriate during the recovery from a surgery, two weeks after the surgery they were housed in groups of four with water and food *ad libitum*, under an inverted dark-light cycle (12:12 h). The animals were 1 year old at the beginning of the experiment (400g—500g of body weight). Gait analysis, rotarod performance, open field and EMG activity were tested before the lesion as initial parameters (normal condition) of the motor coordination, postural balance and muscle rigidity of each rat in order to follow up their performance throughout the experiment. The rats were first tested in the rotarod and trained to remain on the rod at 5 rpm and 10 rpm for 2 min, discarding those that after three consecutive days were unable to stay on the rod; only 40 rats out of 48 met the criterion. Eight of these rats were randomly assigned to an intact group. The remaining 32 rats were injected with 6-OHDA (6-hydroxydopamine hydrochloride; Sigma, St., Louis, MO) into the striatum and after one month the rats that did not reduce their rotarod performance [35] more than 50% were discarded. Only 24 rats fulfilled this criterion, they were divided into three groups of 8 rats each one: 1) treated with the D3 receptors agonist associated with the transfection of BDNF gene (7-OH-DPAT + BDNF), 2) treated with the D3 receptor agonist (7-OH-DPAT), 3) treated with saline. Some animals died for several reasons. The deaths were aleatory and occur throughout the 12 months of the experiment. Apparently, they were not associated with the experimental procedure because it included intact rats. Finally, the groups were as follows: 7-OH-DPAT + BDNF (*n* = 5), 7-OH-DPAT (*n* = 5), Saline (*n* = 6) and intact (*n* = 6). Fig. 1 shows the experimental design. The treatment (7-OH-DPAT infusion plus the BDNF transfection) started 5 months after the unilateral nigrostriatal lesion, when rats were middle age (17-months-old). Motor performance tests were evaluated every 6 weeks during of the treatments (4½ months,) and two months after the end of the D3 agonist or saline infusion (end of treatment), to test the persistence of the effects. It is important to note that the BDNF expression persist until the time of sacrifice. The Immunohistochemical analysis was carried out at the end of the 2 months after treatment when rats were 2 years old.

### 6-OHDA Lesion

The dorso-medial intra-striatal injection sites were chosen in an effort to avoid the nonspecific chemical damage to the medium-sized spiny neurons produced by 6-OHDA [36] in the dorso-lateral striatum, which could, potentially, prevent the reinervation of this area, predominantly receiving afferencies from the sensorimotor cortex [37,38]. Anesthetized rats were mounted on a stereotaxic apparatus (Kopf model 201025R), then 6-OHDA was slowly injected (1μL/3 min) via a 30-G injection needle connected through a polyethylene tube to a 10 μL Hamilton syringe driven by a MicroSyringe Pump Controller (World Precision Instruments). The neurotoxin was injected into two sites (10 μg/μL/site) in the right striatum according with the following coordinates: AP = +1.4, ML = +2.2, DV = -5.2; AP = +0.9, ML = +3.0, DV = -4.2. The coordinates AP and DV were from Bregma according to the Atlas of Paxinos [39] and DV was from the dura mater. The 6-OHDA dissolved in saline solution containing 0.1% ascorbic acid was kept on ice (4°C) and protected from light to minimize oxidation. After ending the injection, the needle remained in its place for 5 min to prevent reflux and to allow for diffusion of the toxin. Once the scalp wound was sutured, the rats were housed individually, and received Terramycin (Pfizer) 2 g/L dissolved in the drinking water for two weeks.

### Administration of the D3 Agonist

The D3 agonist 7-OH-DPAT (Sigma, St., Louis, MO) was systemically administered [40] (0.1 mg/kg/day) via osmotic pumps (Alzet micro-osmotic pump, model 2006) implanted in the abdominal cavity. The pumps were implanted under chloral hydrate anesthesia (300 mg/kg, i.p.) and aseptic conditions. The D3 agonist was infused during 4 1/2 months, replacing the pumps every 1 ½ months. The treatment started when the rats were 17 months old and finish when they were 21 ½ months old.

### BDNF-flag transfection by NTS-polyplex

The plasmid phDAT-BDNF-flag (10.511 kbp) that codes for BDNF-flag was transfected into the dopamine neurons using the neurotensin (NTS)-polyplex nanovector [41]. This transfection is highly selective for dopamine neurons because the NTS-polyplex nanovector is endocytosed via the high affinity neurotensin receptor present only in the dopamine neurons [3,42–44], and also because the BDNF-flag gene expression is under the control of the human dopamine transporter gene promoter (hDAT) [3,45,46]. The surgical procedure for the transfection was as that for the 6-OHDA injection. The NTS-polyplex (2 μL of the phDAT-BDNF-flag/NTS-carrier solution) was injected slowly (0.1 μL/min), just above (in the border) of the substantia nigra pars compacta to avoid the mechanical lesion produced by the injection needle. The injection site was localized at the following coordinates: AP = -5.0, ML = +1.9, DV = -7.0 as described earlier [41]. The coordinates AP and DV were from Bregma according to the Atlas of Paxinos [39] and DV was from the dura mater. Retention and retardation micro assays [42,44] were used to determine the optimum molar ratios of NTS-polyplex components, which were 30 nM plasmid DNA; 30 μM karyophilic peptide; and 1.17 μM NTS-FP-PLL (a conjugate of NTS, a fusogenic peptide and poly-L-lysine) [44,45]. For these molar ratios, the NTS concentration was 1.17 μM, as determined previously with ^125^I-NTS [45]. Taking into account this NTS concentration and the injection volume (2 μL), the NTS-poliplex dose at the time of dosing was 2.34 pmol for rats of 550 g of mean body weight. In accordance with the amount of plasmid DNA, the dose was 419.6 ng of phDAT-BDNF-flag.

### Immunohistochemistry

Twelve months after the 6-OHDA lesion and under chloral hydrate anesthesia (300 mg/kg, i.p.), the rats were perfused transcardially with 120 ml of 0.9% saline, followed by 300 ml of 4% paraformaldehyde. After that, the brains were removed and stored at 4°C in 30% in sucrose for cryoprotection. Slices (50-μm) from the whole brain were obtained using a freezing sliding microtome (Leica; Heidelberg, Germany), and collected in phosphate-buffered saline (PBS) pH 7.4. To deplete endogenous peroxidase, the slices were incubated in a peroxideisopropanol solution (0.3%–10% in PBS) for 30 min at room temperature (RT). Slices were permeabilized with a solution of 0.5% Triton X-100 in PBS 4 times for 10 min at RT. To block unspecific binding sites, slices were incubated in a solution of PBS containing 0.5% Triton X-100 and 10% normal goat serum for 1 hour at RT. Immediately, the slices were incubated with a rabbit anti-tyrosine hydroxylase (TH) polyclonal antibody (1:1000 dilution; Chemicon International; Temecula, CA, USA) for 48 h at 4°C. Next, the slices were washed three times with PBS and incubated with a goat anti-rabbit IgG biotinylated (1:200 dilution; Vector Laboratories; Burlingame, CA, USA) for 2 h at RT. The peroxidase enzyme was detected with diaminobenzidine using the VectaStain Elite kit (Vector Laboratories). The nigral tyrosine hydroxylase-positive (TH+) neurons were visualized with a microscope (Leica DM1000LED) using a digital DC300 camera (Leica; Cambridge, UK) at 10x. The digital images of the substantia nigra pars compacta were projected onto a monitor screen. The substantia nigra pars compacta was delimited considering the following anatomical references: the accessory optic tract in the medial flank, the cerebral peduncle basal part in the lateral flank, the subparafascic nucleus in the superior side and by pars reticulate in the inferior side [39]. The TH+ cells were counted within this area, both in the intact and injured side, using the Qwin Pro Program (Leica Imaging System Ltd.; Cambridge, UK). In our analysis, we applied the principles of the classical stereology technique, but instead of representative sections of the plane, we counted the total number of the TH+ neurons in the whole slice, in order to increase the sample and reduce the statistical error. Slices were taken every 200 μm along the rostro-caudal extension of the substantia nigra pars compacta (10 to 12 slices; n = 4 rats per group). This analysis was unbiased because the person in charge of cell counting was blind with respect to the experimental treatment of the rat.

### Optical Densitometry

The TH immunoreactivity was visualized using a microscope (Leica DM1000LED) with 1.25x objective and a digital camera (Leica DFC290HD). The optical density (OD) of the TH-positive fibers in the striatum was estimated analyzing the TH+ images using ImageJ software (NIH; Bethesda, MD, USA http://rsb.info.nih.gov/ij/). We calibrated the OD using built-in functions of the software (http://imagej.nih.gov/ij/docs/examples/calibration/). We converted the TH+ images to 8-bit grayscale, without rescaling. The OD of the striatum was assessed in three areas: dorsomedial, dorsolateral and ventral, in 12–14 slices taken every 200 μm along the rostro-caudal extension of the striatum, from AP = +1.5 to AP = -1.2. The coordinates AP and DV were from Bregma according to the Atlas of Paxinos [39] and DV was from the dura mater. [39]. The OD of substantia nigra pars reticulata was assessed in 10–12 slices along its rostro-caudal extension. The substantia nigra reticulata was defined as the area below the substantia nigra compacta, delimited by the accessory optic tract in the medial side and the cerebral peduncle in the lateral and inferior side [39]. The OD of the corpus callosum was taken as background for the striatum and the anterior pretectal nucleus for pars reticulata. To quantify the shrinkage of the striatum produced by the 6-OHDA, the total area analyzed of the lesioned striatum in pixels was compared with the total area in pixels of the intact striatum. The total area was calculated as the sum of the pixels determined in every slice of that striatum.

### Retrograde Labeling of Nigral Neurons

Tetramethylrhodamine (Rho) conjugated with dextran amine (10,000 MW, lysine fixable; Molecular Probes; Eugene, OR, USA) was injected (1μL; 0.1 μM/μL) into the striatum (AP = +2.0, ML = +1.9, DV = -5.0), as a retrograde fluorescent tracer, seven days before sacrificing the animals. The coordinates AP and DV were from Bregma according to the Atlas of Paxinos [39] and DV was from the dura mater. The fluorescence of Rho was detected with a confocal microscope (Leica TSC SP8) with 20X objective at 568–585 nm excitation—emission wavelengths. After projecting the digital images of the fluorescent-labeled nigral neurons onto a monitor screen, we counted manually the number of TH+ cells showing the retrograde fluorescent tracer in 10–12 slices per rat taken every 200 μm along the rostro-caudal extension of the substantia nigra pars compacta.

### Immunofluorescence

Slices were permeabilized with a solution of 0.5% Triton X-100 in PBS 4 times for 10 min at RT, then incubated for 30 min at 37°C in PBS containing 0.5% Triton X-100 and 10% BSA (to block unspecific binding sites). Then, exposed for 48 h at RT to the rabbit polyclonal anti- tyrosine-hydroxylase antibody (1/1000, Millipore, Billerica, MA, USA), together with the mouse monoclonal anti-flag antibody (1/300, Sigma-Aldrich Co., St. Louis, MO, USA). Then, the slices were incubated for 2 h at RT with the suitable secondary antibodies: CY5 goat anti-rabbit IgG (1/200; Zymed laboratories, San Francisco, CA, USA), and fluorescein (FITC)-conjugated sheep anti-mouse IgG (1/60; Sigma-Aldrich Co., St. Louis, MO, USA). After 5-min washing with PBS, the nigral slices were mounted on glass slides using Vectashield and protected with coverslips. The fluorescence was detected using a confocal microscope (Leica, TSC SP8). Ten to twelve slices taken every 200 μm along the rostro-caudal extension of the sustantia nigra pars compacta were scanned (1 μm optical thickness, total 30–35 μm) in the z-series, projecting the integrated images onto a two-dimension plane. The fluorescent images were overlapped on the screen monitor using the color green for fluorescein, red for Rho, and blue for CY5. Negative controls were obtained by omitting the primary antibody. The number of fluorescent nigral neurons were counted manually using the Confocal Assistance Program (Leica Confocal Systems, TCS SP8).

### Golgi Method

A block of about 4 mm from the anterior lobe of the brain containing the most rostral part of the striatum (AP = +1.5 to AP = +2.2), very close to the first 6-OHDA injection site, was cut. The bock was immersed into a Golgi solution (potassium dichromate 2.7% and osmium 0.3%) for 7 days, followed by an immersion into 0.75% aqueous solution of silver nitrate for 2 days. Slices (100-μm-thick) were obtained, dehydrated in ethanol (96% and 100%), and after clearing (eugenol and xylol), they were mounted on entellan-covered slides. We counted the dendritic spines along a 10 μm-length of five secondary dendrites out of 10 spiny neurons of each striatum [47,48]. The counting was unbiased because the person counting the spines was blind to the experimental condition of the rat.

### Gait Analysis

**Fig 2:**
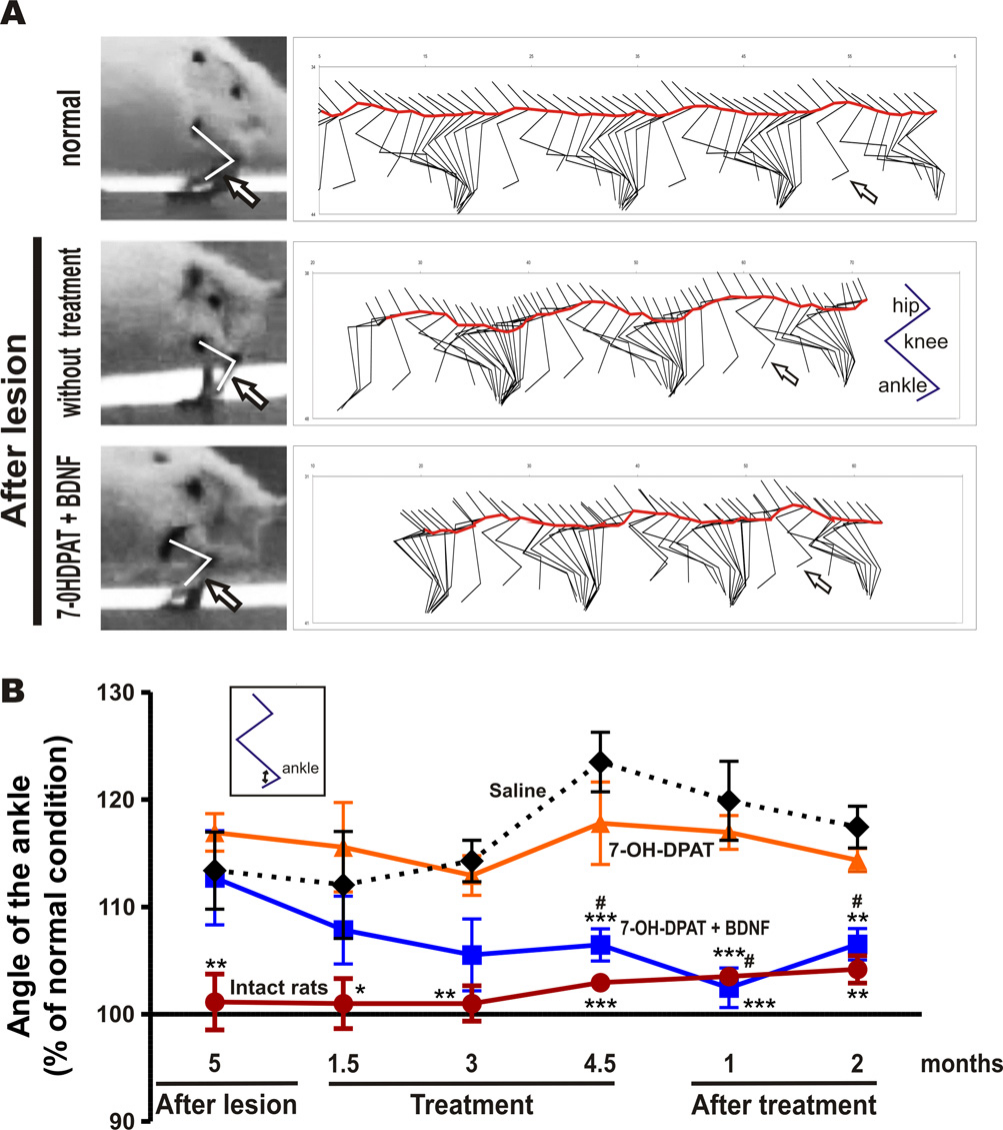
The combined treatment restored gait. The lesion increased the angle of the contralateral ankle during the dorsiflexion of the swing phase of gait (arrows) and the undulatory displacement of the hip (limping), red line in A. The combined treatment normalized both conditions (A, B) and supplementary videos (S1 Video—GAIT ANALYSIS IN NORMAL SPEED and S2 Video—GAIT ANALYSIS IN SLOW MOTION). The D3 agonist alone did not restore neither the hip displacement (not shown) nor the angle of the ankle during gait. The increase in the angle produced by the lesion persisted all along the experiment (saline-treated rats) in B. The recovery produced by the combined treatment was significant (F_3, 15_ = 26.09, *P* < 0.0001, One-way repeated-measures ANOVA). *P < 0.05; ** *P* < 0.01; *** *P* < 0.001compared with saline-treated rats; ^#^ *P* = No significant difference compared with intact rats (Bonferroni’s Multiple Comparison Post tests). A, illustrate a representative case. The data are given as the mean ± SEM (*n* = 5–6 rats per group).

Gait disorder is one of the most incapacitating symptoms of *Parkinson’s disease*. Gait analysis in rodent models of Parkinson´s disease allows testing the degree of loss of dopamine in striatum [49]. Here we studied gait to assess the recovery of the dopamine innervation of the striatum by the employed treatments. Gait was recorded on a transparent acrylic runway (17 cm- high, 15 cm wide and 170 cm long, with a dark compartment at the end of the runway for sheltering). The runway was 150 cm above floor level. The iliac crest, greater trochanter, lateral malleolus, and the fifth metatarsal distal head were marked with indelible ink, as reference for analysis. To avoid inter-tester variability, the same operator evaluated gait throughout the experiment. As the gait analysis was limited to the sagittal plane, the camera was placed facing the left side of the rat (contralateral to the 6-OHDA lesion), perpendicular to the direction of movement. The marked points were tracked frame by frame, obtaining a two-dimensional coordinates (x, y) using the ImageJ software (NIH; Bethesda, MD, USA http://rsb.info.nih.gov/ij/). Data were imported into Microsoft Office Excel 2010 (Microsoft) and further analyzed with pre-assembled Excel sheets to modeled body segments as rigid straight lines between the marked points. The knee position was computed indirectly by superimposing two circles centered on hip and ankle pivots, with a radius of the length of the femur and tibia respectively [50]. The kinematics of gait was reconstructed from changes in the marked points located between consecutive frames, facilitating the generation of stick diagrams (superimposing modeled body segments of every frame) and spatial displacement plots. Angles and distances were calculated directly by the software. For angle measurements, we used the smaller angle of the two alternatives (insert Fig. 2B); typically, this was the angle at the flexor side of the joint. We chose to analyze the angle of the ankle joint because it was the one most altered by the 6-OHDA lesion. To learn to move along the runway with constant speed, the rats were trained during three consecutive days before the recording session. One session usually consisted of 10 passes along the runway. Four satisfactory strides per rat were obtained when the rat moved with relatively constant speed (center of the runway). Optical deformation of the image produced by the camera lens was determined and corrected by using an acrylic square (5 cm x 5 cm) which served as a bi-dimensional scale.

### Rotarod Performance

This test evaluates motor coordination and balance as well as motor learning. In the rat, both aspects are under the control of the dopaminergic innervation of the dorsal striatum: the dorsomedial part controls motor learning and the dorsolateral part controls motor coordination and balance [37,51–53]. Motor performance of rodents in the rotarod allows for the evaluation of the loss or recovery of nigrostriatal innervation in rodent models of Parkinson´s disease [35,54]. The rotarod consists of a four-lane rotating rod (diameter 7.5 cm.) and infrared beams to detect the moment of fall. The body of the rat was placed perpendicular to the rotating axis and the head against the direction of the rotation; the animal must therefore move forward to stay on the rod. Since several rats were generally tested in the same session, each rat rested for about 15 min between the different testing speeds, thus reducing stress and fatigue. The rats were trained twice on the rotarod at the constant speed of 5 and 10 rpm for two min during three consecutive days before the evaluation of their performance. In the evaluating session, the rats were placed on the rod and their performance was tested at different constant speeds (5, 10, 15, 20 and 25 rpm) for a maximum of 2 min at each speed. All rats were video-recorded while staying on the rod to assess the qualitative aspect of recovery of motor coordination and posture.

### Open Field Test

The evaluation of the behavior of the rats in the open field was performed approximately every 6 weeks. The rat was placed in the center of a square arena (80 × 80 cm) with 40 cm high, opaque black walls, in a quiet, red light illuminated room. The motor behavior was video recorded for 20 min. The geometrical coordinates of the rat position in the arena were measured frame by frame from the recorded videos to obtain the spatiotemporal sequence of the movements. The following behavioral parameters were measured in the first ten minutes in the open field: walking distance (ambulation), rearing and walking speed. Bradykinesia was estimated by the reduction in walking speed assessed by the time taken by the rat to move from one corner of the open-field to the next one with relative constant speed. The arena was cleaned with a water/alcohol (70%) solution before every behavioral testing to avoid a possible bias due to odors and/or residues left by rats tested earlier. All experiments were carried out from 11:00 a.m. to 3:00 p.m.

### EMG Recording

The EMG was recorded from the surface of the gastrocnemius and tibialis anterior muscles using a pair of stainless steel electrodes (Grass electrodes) inserted subcutaneously. The subcutaneous location of the electrodes avoids damaging the muscle structure. A fifth wire (ground) was also placed subcutaneously close to the recording ones. The EMG signal was acquired with an amplifier DAM 80 (World Precision Instruments, amplification factor of 1000, filtered at 1 Hz—10 kHz). The resultant signal was recorded and digitized at a sampling rate of 100 Hz with an oscilloscope (Agilent 54622A). EMG is expressed as mean tonic EMG activity (mV/20s). To avoid the activation of muscle and tendon receptors, the rat was suspended with the aid of an adjustable soft leather jacket, which covered the head, thorax, forelimbs and the base of the rear limbs. The rats were generally quiet during the recording and there was no phasic bursting EMG activity due to joint movements. Tonic EMG activity was recorded when the animal was completely relaxed but awake. The environment was always kept quiet and dark.

### Data Analysis

Gait, rotarod performance, open field behavior and EMG activity analysis from each rat were analyzed before the 6-OHDA lesion. The values of these analyses were taken as 100% (normal condition). The values obtained in subsequent evaluations were expressed as percentage of the values of the normal condition of the same animal, to rule out the variability among animals of the same species and age. Values are provided as the mean ± SEM. As the same animals were tested multiple times, the sequential effects of treatments and the differences among groups were estimated by repeated measures one-way ANOVA test and Bonferroni´s multiple comparisons post-test. In the histological analysis, statistical significance was determined by one-way ANOVA followed by Bonferroni´s multiple comparisons post-test. Statistical significances were estimated using GraphPad Prism version 5.0 program (GraphPad Software, San Diego California, USA). *P* < 0.05 was taken as a statistically significant difference.

## Results

### Effect on Gait

The loss of dopamine in the rodent model of Parkinson´s disease increases the angle of the ankle during the swing phase of gait [55,56], which indicates reduced dorsiflexion of the ankle. Here the unilateral dorsomedial striatal lesion with 6-OHDA increased the angle of the ankle of the contralateral hind limb by about 16% at the end of the flexion during the swing phase of gait (arrows in Fig 2A, middle stick diagram). The administration of the D3 agonist associated with the BDNF transfection progressively normalized the angle of the ankle (arrows in Fig 2A, lower stick diagram). The recovery persisted at least two months after the end of the combined treatment (Fig. 2B), suggesting a trophic effect of the treatment. The lesion also produced a limping of the contralateral limb, which was expressed by an increase in the hip undulating displacement during gait (red line in Fig. 2A). The D3 agonist associated with the BDNF transfection practically eliminated the limping and normalized the hip displacement (Fig. 2A and S1 Video- Gait analysis and S2 Video- Gait analysis in slow motion). By contrast, the D3 agonist alone did not normalize the angle of the ankle (Fig. 2B) or the hip displacement (not shown). The lesion also increased the stance phase of gait by about 17% and reduced the flexion angle of the knee by about 12%; the combined treatment significantly (*P* < 0.05) restored to normal both conditions (not shown). The lesion did not affect walking speed or step length (not shown).

### Effect on Motor Coordination

**Fig 3:**
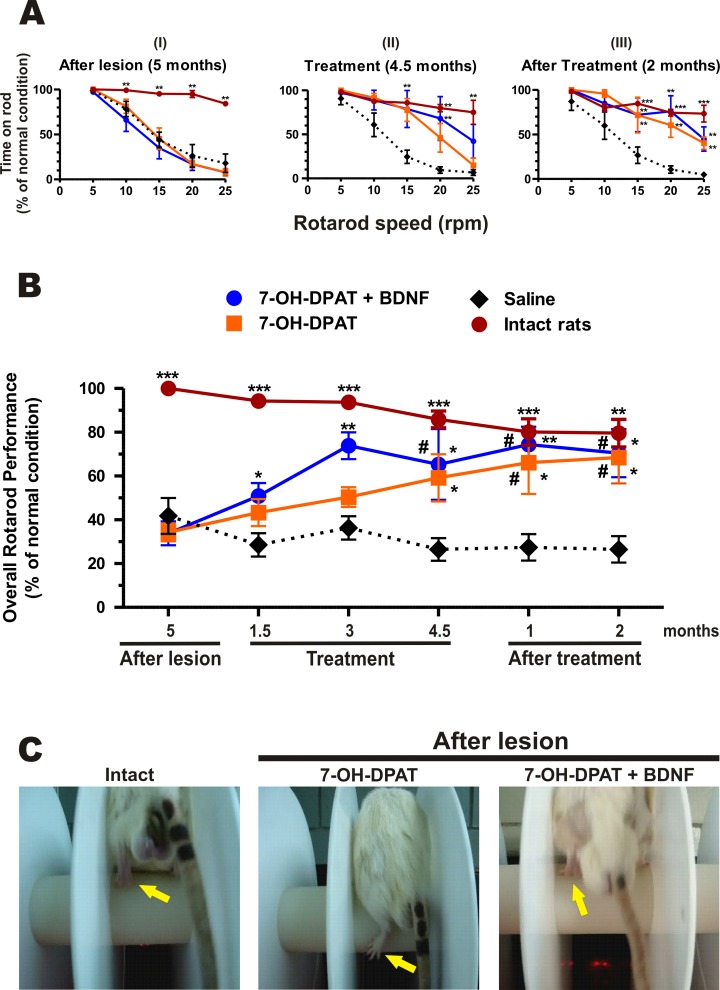
Administration of the D3 agonist associated with the BDNF transfection restored motor coordination. The lesion reduced the time on the rod (A-I) and the rotarod performance (B). The combined treatment normalized the time on the rod (A-II) and the rotarod performance (B). The effect persisted two months after the treatment (A-III and B). The D3 agonist alone also recovered the motor coordination (A, B) but the rats dragged the contralateral hind limb during rod rotation (arrows in C and supplementary videos S3 Video—ROTAROD PERFORMANCE IN NORMAL SPEED and S4 Video- ROTAROD PERFORMANCE IN SLOW MOTION). Saline-treated rats did not recover the rotarod performance (A, B). The recovery produced by the treatments were significant (AI: F_3, 12_ = 9.881, *P* < 0.0015; AII: F_3, 12_ = 8.879, *P* < 0.0023; AIII: F_3, 12_ = 12.49, *P* < 0.0005 and B: F_3, 15_ = 21.84, *P* < 0.0001, One-way repeated-measures ANOVA). * *P* < 0.05; ** *P* < 0.01; *** *P* < 0.001 compared with saline-treated rats. ^#^ *P* = No significant difference compared with intact rats (Bonferroni’s Multiple Comparison Post tests). The data are given as the mean ± SEM (*n* = 5–6 rats per group). C illustrates representative cases.

The motor coordination and balance of the rats was evaluated by the amount of time that the rats remained on the rod at the different constant rotation speeds (Fig. 3A) and by their overall rotarod performance (Fig. 3B), calculated as the area under the curve in a plot of time-on-the-rod vs. rotation speed [35,54]. The D3 agonist associated with the BDNF transfection as well as the D3 agonist recovered the motor coordination and balance (Fig. 3A and B). The recovery by the combined treatment was faster than that of the D3 agonist alone. After 1 ½ months of the combined treatment the rotarod performance of the treated rats was significantly different from that of the saline treated rats, and after 4 ½ months their rotarod performance was not statistically different from that of intact rats (Fig. 3B). The sole administration of the D3 agonist also recovered the rotarod performance, but the recovery was slower and not statistically different from that of normal rats until the end of treatment. However, the recovery was not as complete as the combined treatment: the rats treated with the sole D3 agonist dragged the contralateral hind limb while staying on the rod (Fig. 3C and S3 Video- ROTAROD PERFORMANCE and S4 Video—ROTAROD in slow motion).

### Effect on Muscle Rigidity

**Fig 4:**
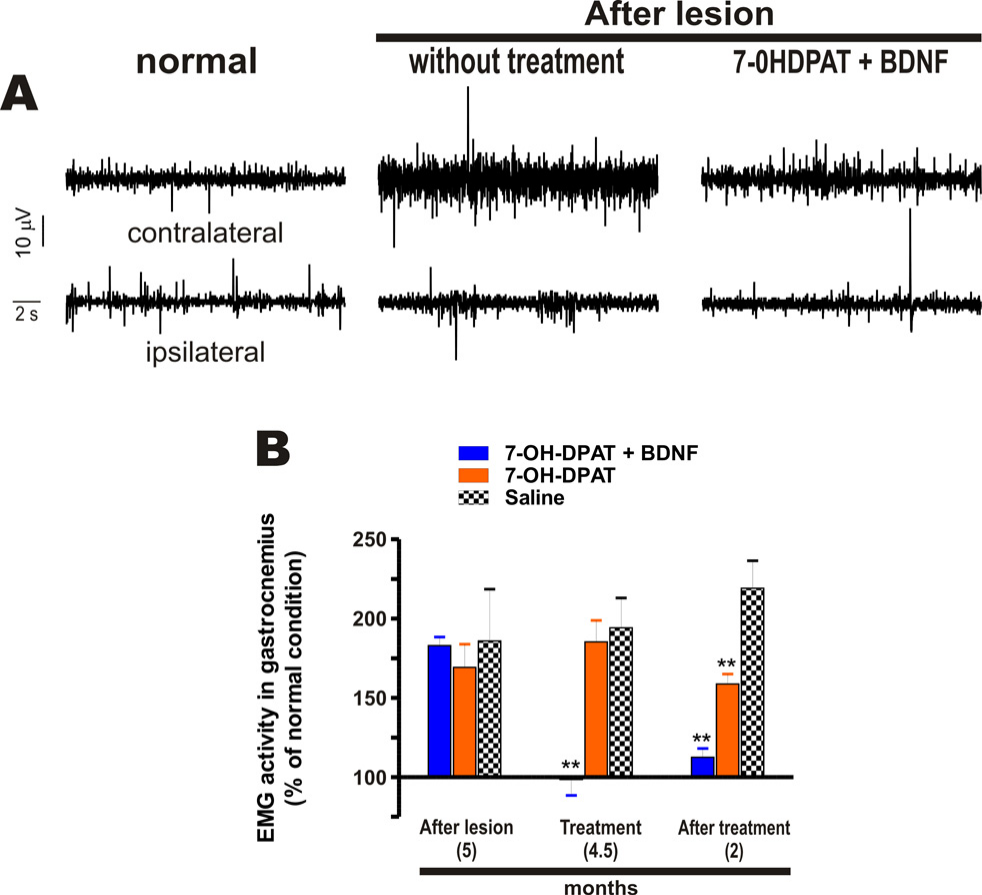
The combined treatment recovered normal muscle tone. The lesion increased the EMG activity of the contralateral gastrocnemius but did not affect the EMG of the ipsilateral one (A). The combined treatment normalized the EMG activity (A and B) and the effect persisted two months after the treatment (B). The D3 agonist also reduced the EMG, but the reduction was partial and occurred two months after the treatment (B). The recovery produced by the combined treatment was significant (F_2, 6_ = 5.674, *P* < 0.0414, One-way repeated-measures ANOVA). * *P* < 0.05; ** *P* < 0.01 compared with saline-treated rats (Bonferroni’s Multiple Comparison Post tests). The data are given as the mean ± SEM (*n* = 5 rats per group). A, shows a representative case.

The loss of the dopamine neurons, both in experimental and human Parkinson´s disease, produces muscle rigidity. Here we assessed muscle rigidity by the increase in electromyographic (EMG) activity of the muscles of the hind limb. The lesion increased the EMG activity of the contralateral gastrocnemius but not of the ipsilateral one (Fig. 4A). In addition, the lesion did not consistently affect the EMG activity of the tibialis anterior of either side (not shown), suggesting that the loss of dopamine produced rigidity mainly of extensor muscles. The D3 agonist associated with the BDNF transfection normalized the increased EMG activity of the contralateral gastrocnemius (Fig. 4A), and the EMG activity remained normal after two months of the end the treatment (Fig. 4B). The D3 agonist alone also recovered the EMG activity, but the recovery was partial and occurred two months after the end of the treatment (Fig. 4B). The saline-treated rats did not recover the normal EMG activity at any moment of the treatment (Fig. 4B), indicating that there was no spontaneous recovery of the muscle tone.

### Effect on Motor Behavior in the Open Field

**Fig 5:**
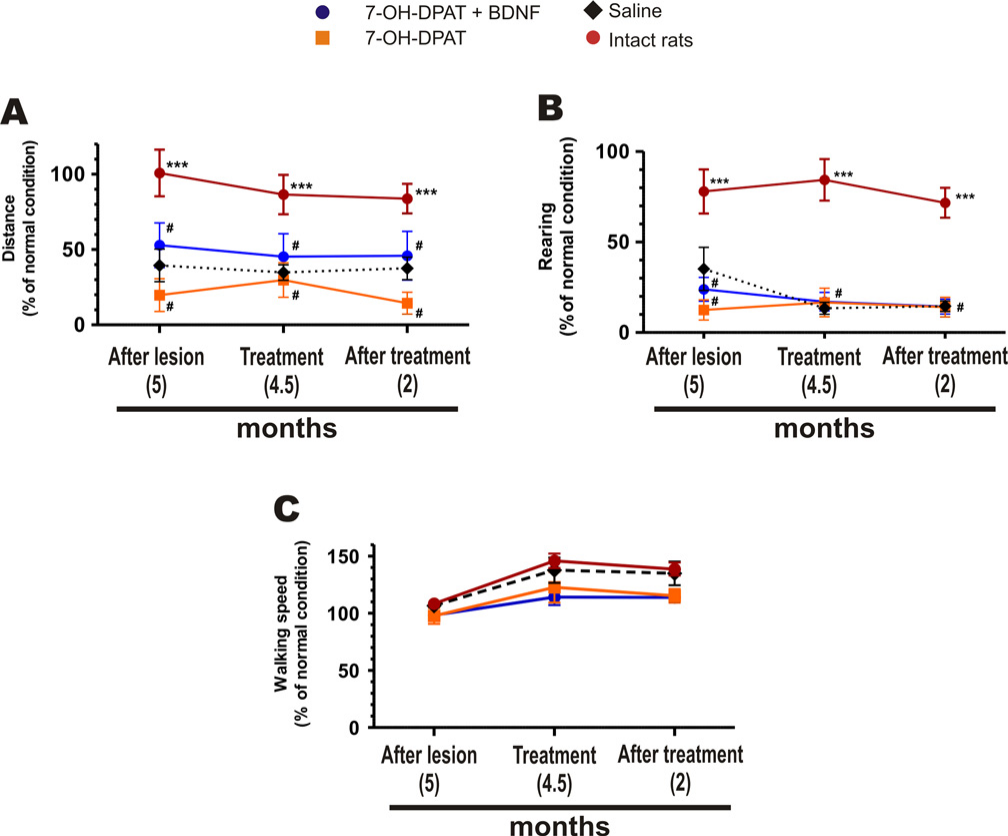
The combined treatment did not recover the ambulatory activity and rearing in the open field. The lesion reduced the ambulatory activity evaluated by the distance traveled (A) and frequency of rearing (B) during the first ten min in the open field. However, it did not produce bradykinesia assessed by the speed of walking (C). None of the treatments recovered the ambulatory activity or rearing (A and B). Intact rats did not showed changes either in ambulation or in rearing, throughout the experiment. The 6-OHDA lesion reduced significantly the traveled distance and frequency of rearing (A: F_3, 6_ = 73.75, *P* < 0.0001 and B: F_3, 6_ = 57.30, *P* < 0.0001, One-way repeated-measures ANOVA) but it did not produce a significant effect on bradykinesia (C: F_3, 6_ = 3.563, *P* < 0.0868, One-way repeated-measures ANOVA). *** *P* < 0.001 compared with saline-treated rats. ^#^ *P* = No significant difference compared with saline-treated rats (Bonferroni’s Multiple Comparison Post tests). The data are given as the mean ± SEM (*n* = 5–6 rats per group).

The unilateral lesion reduced the locomotion of the rats in the open field (Fig. 5). The reduced locomotion could be either by bradykinesia or by an increase in anxiety. Because rats were not habituated, due to the prolonged interval between testing, the open field was a novel environment that possibly triggered anxiety in the rats. To decide between the bradykinesia and anxiety, we determined the speed of walking to assess bradykinesia, and the ambulation and frequency of rearing during the first ten min in the open field to assess anxiety [57–59]. As seen in Fig. 5, the lesion did not produce bradykinesia but reduced ambulation and the frequency of rearing, suggesting that the reduction was mainly due to an increase in anxiety. None of the treatments was able to normalize ambulation or rearing (Fig. 5A and B). The intact (normal) rats maintained their initial ambulation and rearing (Fig. 5), indicating that there was no habituation because of the repeated exposures to the open field, probably because the evaluations were performed every 1 ½ months, a time enough to rule out habituation. The reduced ambulation and frequency of rearing remained reduced throughout the experiment in the saline-treated rats, indicating that there was no spontaneous recovery.

### BDNF-Flag Expression

**Fig 6:**
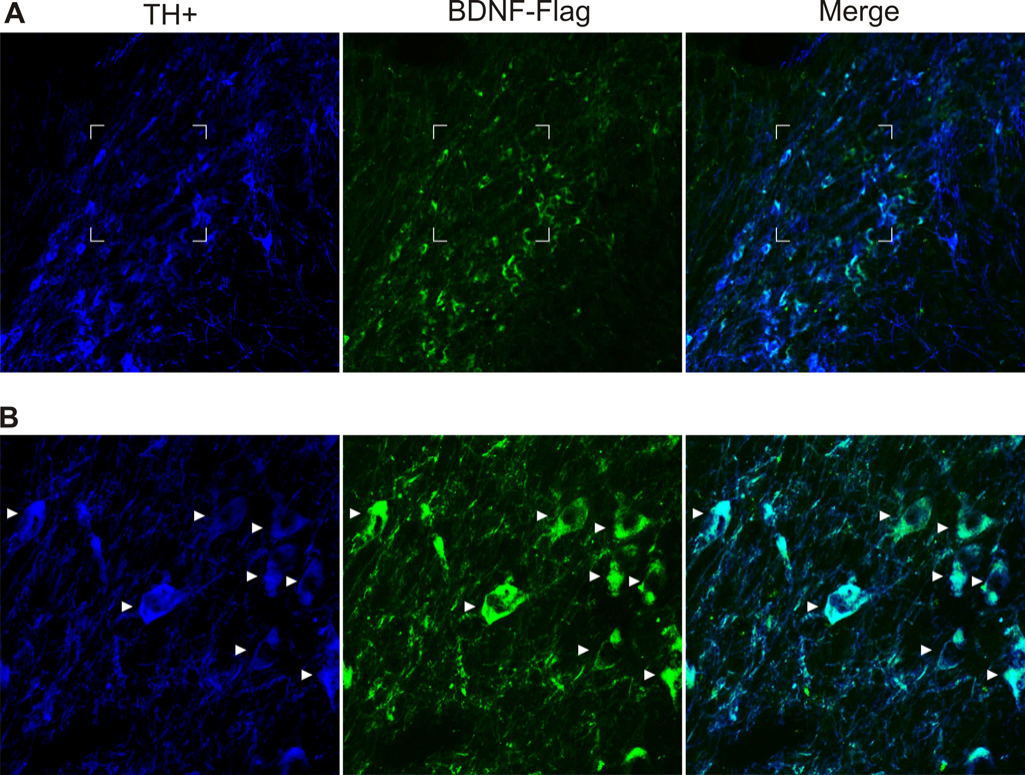
BDNF gene transfection by NTS-polyplex to dopamine neurons of substantia nigra pars compacta. (A) Panoramic view of the labeled TH+ neurons. (B) Amplification of the zone indicated by the frame in A. The arrowheads show the co-localization of the BDNF-flag in the TH+ neurons. This is a representative illustration of the BDNF-flag expression.

In a previous study [45], we showed that the transfection BDNF-flag gene by the NTS-polyplex to TH+ neurons produced a high expression of the BDNF-flag in the dopamine neurons of the pars compacta. Both the injection of the NTS-polyplex in the striatum (retrograde transfection) and the injection of the NTS-polyplex in the substantia nigra (direct transfection) resulted in a high BDNF-flag expression. Here we also found that the BDNF gene transfection by the NTS-polyplex produced a high BDNF-flag expression in the dopamine neurons of the pars compacta, as indicated by the co-localization of the TH-immunoreactivity with the BDNF-flag immunoreactivity in the same dopamine neurons. About 80% of the nigral neurons showed the co-localization (Fig. 6).

### Recovery of TH+ Nigral Cells

**Fig 7:**
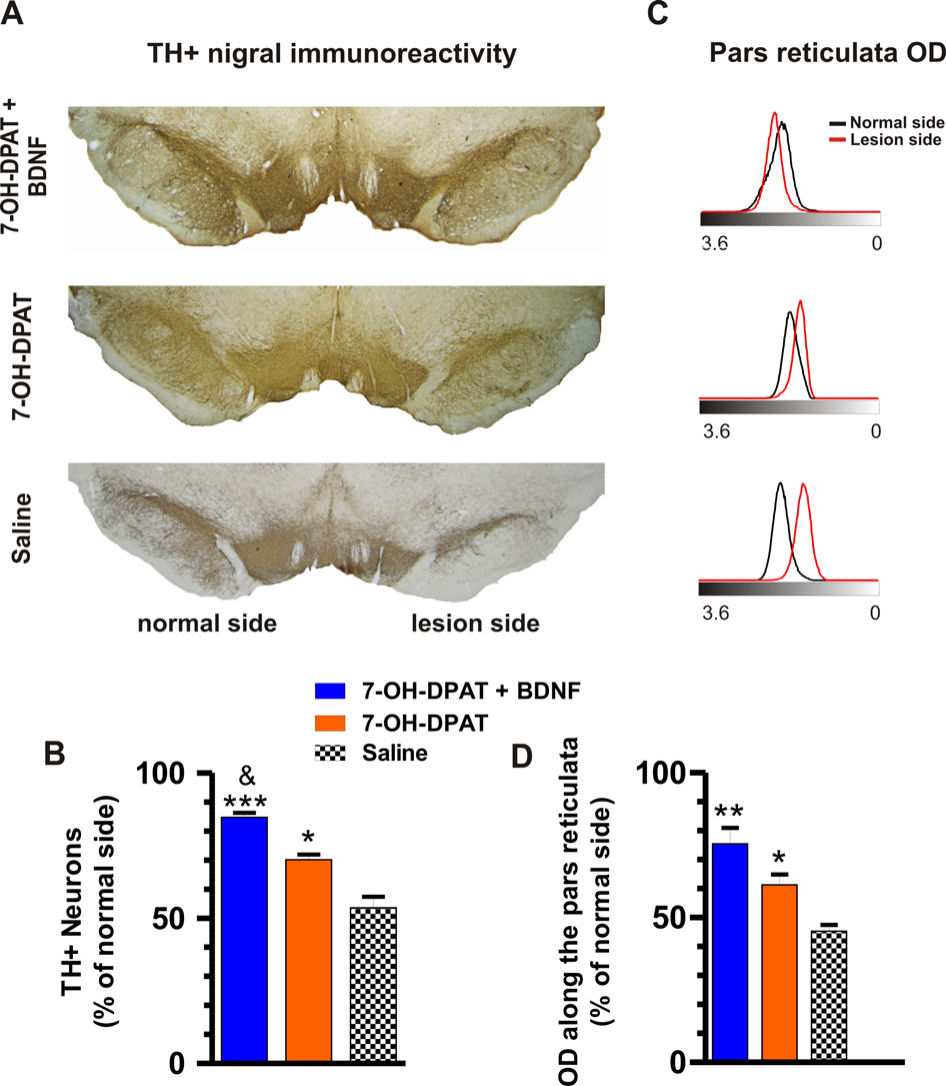
The combined treatment restored the number of dopamine neurons of the sustantia nigra pars compacta. (A) Representative microgaphs of TH-immunoreactivity in mesencehalon slices. (B) Number of TH+ neurons along the rostro-caudal extention of the susbtantia nigra pars compacta. (C) Optical densitometry analysis of TH-immunoreactivity in the substantia nigra pars reticulata of the micrographs in A showing that the combined treatment recovered the dopamine dendrites, judged by the overlapping of the lesion side histogram (red) with the normal side histogram (black). (D) Graph of the OD along the pars reticulata under the different treatments. The recovery produced by the treatments was significant (B, F_2, 8_ = 56.00, P < 0.0001; D, F_2, 9_ = 26.66, *P* < 0.0002, One-way ANOVA). *P < 0.05, **P < 0.001 compared with saline-treated rats, ^&^ *P* < 0.01 compared with the 7-OH-DPAT alone (Bonferroni’s Multiple Comparison Post tests). The data are given as the mean ± SEM (*n* = 4). A, representative images of the TH-immunoreactivity in substantia nigra pars compacta and reticulata.

The 6-OHDA lesion reduced the number of TH+ nigrostriatal neurons (Fig. 7A and B) and decreased the TH+ dendrites of the pars reticulata, as indicated by the reduction of the optical density of the TH immunoreactivity (Fig 7A and C). The D3 agonist associated with the BDNF transfection as well as the administration of the D3 agonist alone induced a significant recovery of the number of the TH+ cells of pars compacta and dendrites of pars reticulata. However, in both cases the recovery produced by the combined treatment was greater than that of the D3 agonist alone (Fig. 7B and C).

### Recovery of the Striatal Innervation

**Fig 8:**
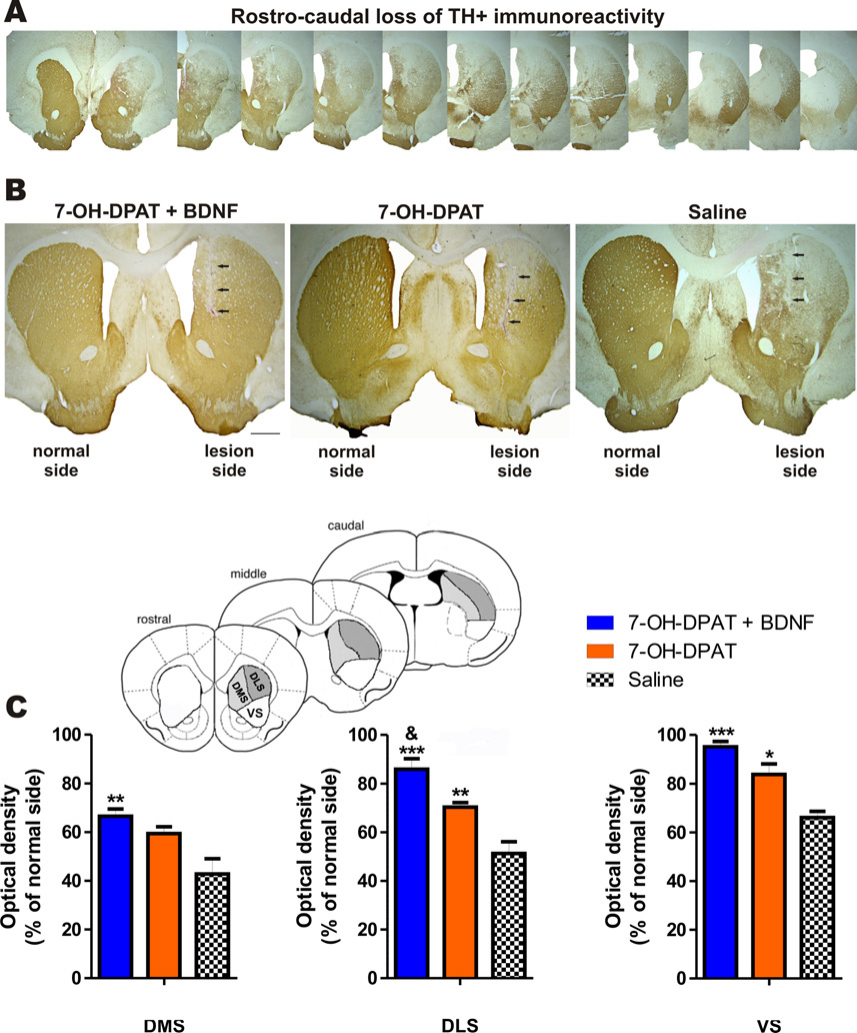
The combined treatment restored the TH immunoreactivity of the striatum. (A) Representative micrographs of TH immunoreactivity showing that the lesion extends along the rostro-caudal extention of the striatum. (B). Representative micrographs showing the effect of the treatments on TH immunoreactivity of the striatum. The black arrows show lesion produced by 6-OHDA. (C) Optical densitometry analysis of TH-immunoreactivity in dorso-medial (DMS), dorso-lateral (DLS) and ventral (VS) regions of the striatum. The recovery produced by the treatments was significant (DMS: F_2, 9_ = 9.694, *P* < 0.0057; DLS: F_2, 9_ = 40.46, *P* < 0.0001 and VS; F_2, 9_ = 19.93, *P* < 0.0005, One-way ANOVA) * *P* < 0.05; ** *P* < 0.01; *** *P* < 0.001 compared with saline-treated rats; ^&^ *P* < 0.01 compared with the 7-OH-DPAT alone (Bonferroni’s Multiple Comparison Post tests). The data are given as the mean ± SEM (*n* = 5 rats per group). The insert shows the three different sectors where the OD was determined.

The recovery of the innervation of striatum was assessed by the TH-immunoreactivity. The 6-OHDA lesion produced a shrinkage of the striatum that varied from 4 to 20% (mean 14%) compared with the intact side. The lesion also produced a marked loss of TH-immunoreactivity in the dorsomedial, dorsolateral and ventral striatum all along its rostro-caudal extension (Fig. 8A and C). The loss tended to be greater in dorsomedial striatum (Fig. 8C). The combined treatment as well as the treatment with the D3 agonist alone restored the TH-immunoreactivity, but the combined treatment recovered the immunoreactivity more fully than that of the agonist alone (Fig. 8B and C).

**Fig 9:**
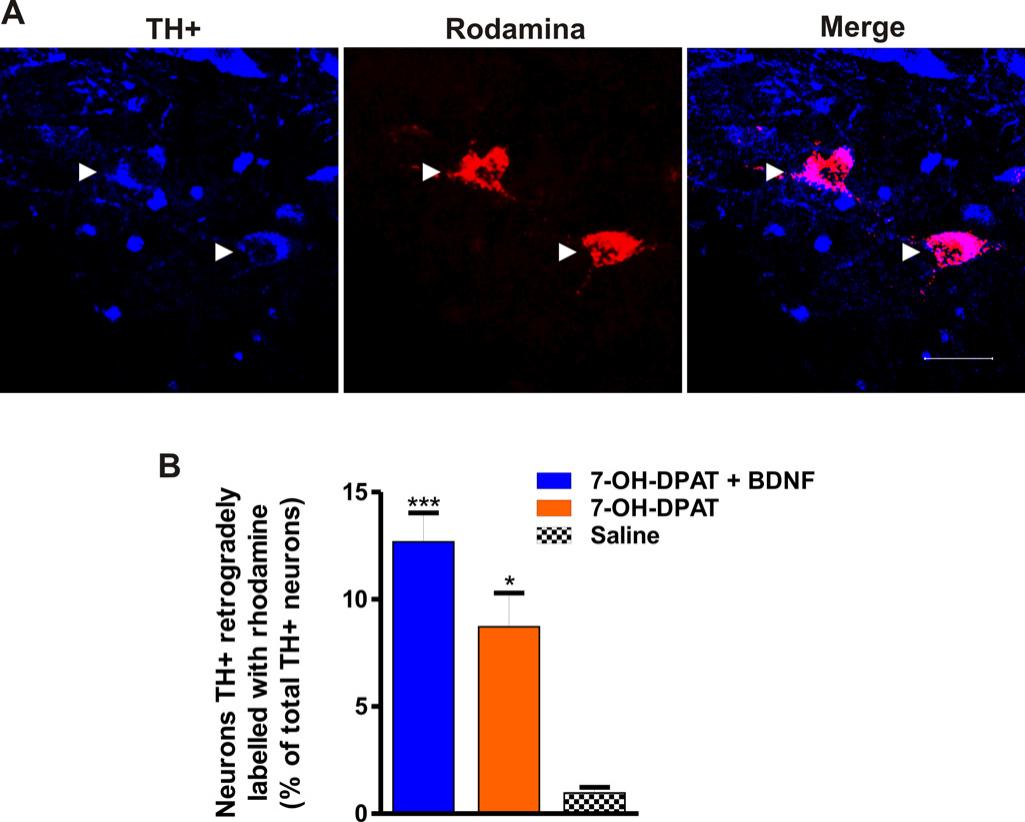
The combined treatment recovered the nigro-striatal innervation. (A) Representative micrographs of the substantia nigra pars compacta showing TH+ neurons with the presence of rhodamine-labeled dextran amine (arrowheads), previously injected into the striatum. (B) Graph showing the percentage of rhodamine-labeled TH+ neurons with respect to the total of TH+ neurons of the same substantia nigra pars compacta, in each experimental condition. The recovery produced by the treatments was significant (F_2, 8_ = 18.34, *P* < 0.0010, One-way ANOVA). * *P* < 0.05; ** *P* < 0.01 compared with saline-treated rats (Bonferroni’s Multiple Comparison Post tests). The data are given as the mean ± SEM (*n* = 4 rats per group).

The recovery of the striatal dopamine innervation was also estimated by the number of dopamine neurons retrogradely labeled by Rho-dextran amine injected in the striatum (Fig. 9A) [60,61]. The D3 agonist associated with the BDNF transfection significantly increased the number of TH+ cells retrogradely labelled with Rho. The treatment with the D3 agonist also increased the number of cells retrogradely labelled but to a smaller extent compared with the combined treatment (Fig. 9B), suggesting that the latter brings about a greater reinnervation of the striatum. About 14% of Rho+ neurons were not positive for TH.

### Recovery of Dendritic Spines

**Fig 10:**
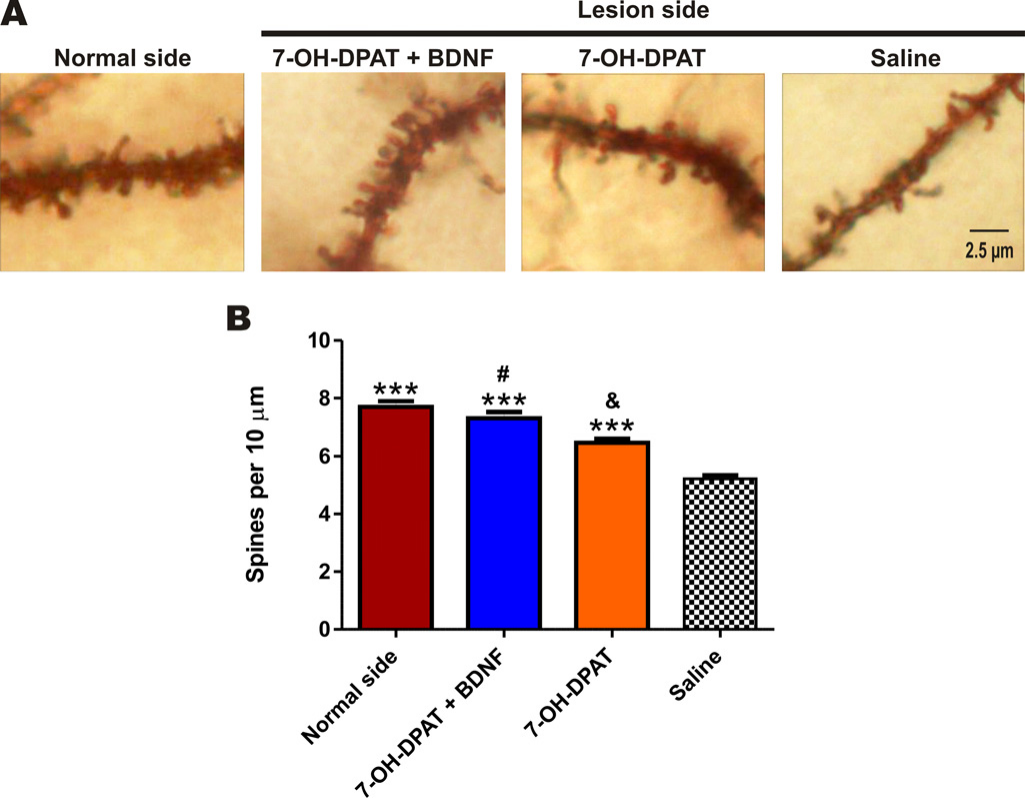
The combined treatment restored the dendritic spines of the medium-sized spiny neurons of the striatum. (A) Representative micrographs of the Golgi-stained spines of the medium-size spiny neurons under the different conditions. (B) Mean number of the spines from 50 striatal secondary dendrites per rat in each experimental condition. The recovery produced by the treatments was significant (F_3, 596_ = 102.1, *P* < 0.0001, One-way ANOVA). ***P < 0.001 compared with saline-treated rats. ^&^ *P* < 0.01 compared with the rats treated with the combined treatment. ^#^ *P* = No significant difference compared with normal side (Bonferroni’s Multiple Comparison Post tests). The data are given as the mean ± SEM. (*n* = 3 rats per group)

The nigrostriatal lesion significantly reduced the number of spines of the medium-sized spiny neurons of the striatum (Fig. 10A and B). The D3 agonist associated with the BDNF fully recovered the spines. The D3 agonist alone produced a significant recover of the number of spines, however, the recovery was partial (Fig. 10A and B).

## Discussion

Because PD is more common in older people, their reduced motor performance is caused by the disease and senescence. Accordingly, older rats might increase the predictive validity of pre-clinical rodent models since they exhibit more robust parkinsonian motor deficits than younger rats [62]. Age-related degeneration of mesencephalic dopaminergic cells is produced in part by a decreased availability or efficacy of neurotrophic factors [63]. This is why therapeutic approaches with viral vectors are used to increase levels of striatal BDNF or GDNF in aged rats [64,65]. However, age reduces the efficiency (40%–60%) of viral-vector-mediated gene transfer in comparison to young rats [66]. Therefore, in the present study, the nonviral transfection in combination with a D3 agonist treatment was evaluated in a period from the middle aged (17 months) to aged rats (24 months).

Our results show that the transfection of the BDNF gene to dopamine neurons associated with the chronic (4 ½ months) and continuous administration of the dopamine D3 receptor agonist recovers normal gait, motor coordination and postural balance in the unilateral striatal rat model of Parkinson´s disease. A recovery up to 80% of dopamine neurons of substantia nigra and about the same percentage of recovery of the dopamine innervation of the striatum accompanied the recovery of these motor impairments. A total recovery of dendritic spines of striatal medium-sized spiny neurons and a recovery of normal muscle tone also accompanied the recovery of the motor behavior.

Van Kampen and Eckmann [31], administered the dopamine D3 agonist 7-OH-DPAT for up to two months into the third ventricle in rats with unilateral lesions of the nigrostriatal innervation induced by the application of 6-OHDA in the striatum, a model representing early Parkinson´s disease, like the model used here. They chose the preferential dopamine D3 receptor agonist because of its mitogenic role both *in vitro* [25–27,67] and *in vivo* [23,68]. They found that the chronic and continuous infusion of the D3 agonist induced cell proliferation in the substantia nigra pars compacta; many of the cells acquired the dopaminergic phenotype. The treatment reduced amphetamine-induced turning, and recovered the use of the contralateral forelimb for feeding (staircase test). Our results showed that D3 agonist also recovers complex aspects of motor behavior, like motor coordination in the rotarod. However, the recovery was not as complete as when the treatment with the D3 agonist was associated with BDNF transfection. The association normalized gait and abolished muscle rigidity, which the D3 agonist did not. Furthermore, the association showed a greater recovery of the dopamine neurons of the pars compacta, striatal innervation and dendritic spines of the striatal neurons, all of which indicates that the transfection of BDNF to dopamine neurons potentiates the trophic action of the D3 agonist. It remains to be determined whether neurogenesis mediates the recovery of dopamine neurons, as proposed by Van Kampen and Eckman [31].

The rats with unilateral nigrostriatal lesions showed a reduction of about 25% in the number of spines of the striatal medium-sized spiny neurons (Fig. 10), a similar value was found by Solis, et al. [69] in rats with 6-OHDA lesion. In Parkinson´s disease patients studied post-mortem, Zaja-Milatovic et al. [70], reported a reduction of about 45%, whereas Stephens et al. [71], reported a 27% reduction. In the rat with a 6-OHDA unilateral lesion, the reduced number of spines corresponds with a similar reduction in glutamatergic synapsis from cortico-striatal afferents [72], which may explain the motor behavior impairment. Accordingly, the full recovery of the dendritic spines brought about by the activation of the D3 receptors associated with the transfection of BDNF may explain the recovery of motor behavior in the present work. This idea is in line with the fact that the efficacy of striatal dopamine grafts also requires the recovery or preservation of dendritic spines [73].

Double and Crocker showed [74] that the loss of the activation of dopamine receptors in substantia nigra pars reticulata results in muscle rigity, as indicated by the increase in EMG activity of the gastrocnemius and tibialis anterior muscles. In Parkinson´s disease, the dopamine receptors would not be activated because of the loss of dopamine, which would explain the muscle rigidity accompanying the disease. The combined treatment significanlty recovered the dopamine (TH+) dendrites in substantia nigra pars reticulata (Fig. 7C and D), which could have been the source of dopamine to activate the proper receptors in pars reticulata, thus explaining the normalization of the EMG activity produced by the treatment (Fig. 4). The efferent projection of substantia nigra pars reticulata to the mesopontine tegmental area of the brainstem controls gait and muscle tone. The loss of dopamine present in rodent models of Parkinson´s disease markedly increases the inhibitory input from pars reticulata to the locomotor mesencephalic area and pedunculo pontine nucleus, which disturbs gait and increases muscle tone [75,76]. However the effect of the lesion on gait appears to be different depending on whether the 6-OHDA is applied in the striatum or in the forebrain bundle, because the extent of lesion is typically much greater following delivery of 6-OHDA to the forebrain bundle. The lesion reduces the stride length in the first case [77] but there is no reduction in the second case (present results), which is conistent with the finding that the lesion did not produce bradykinesia (Fig. 5). The full recovery of normal gait induced by the combined treatment (Fig. 2) may be associated with the recovery of the normal inhibitory input from pars reticulata to the these brainstem nuclei controlling gait and muscle tone.

The unilateral lesion of the nigrostriatal innervation apparently produces anxiety, assessed by a decrease in ambulation and frequency of rearing in the open field [78]. However, in the present study, the apparently increased anxiety expressed by the decrease in ambulation and frequency of rearing produced by the unilateral striatal lesion of the nigrostriatal innervation was not accompanied by bradykinesia (Fig. 5), which suggests a non-motor cause of the anxiety. Intact rats did not reduce their ambulation and rearing levels indicating that there was no habituation, probably because the evaluations were done every 1 ½ months, a long enough time to rule out habituation. In the present study, none of the treatments was able to eliminate anxiety. Eskow et al., [78] found that the unilateral lesion of the nigrostriatal innervation induced by 6-OHDA injected in the forebrain bundle produced anxiety (reduced ambulation and frequency of rearing) and depression (reduced climbing in the forced-swimming test). They also reported that L-Dopa did not eliminate these manifestations of anxiety. The reason for the failure of the combined treatment and of L-Dopa to control anxiety is unknown.

Physiological expression of BDNF-flag gene could be a key point in the efficacy of the combined treatment. The BDNF-flag gene transfection to dopamine neurons produced the expression of the BDNF-flag protein in a rather large number (80%) of the dopamine neurons of substantia nigra compacta (Fig. 6). However, it is possible that the BDNF-flag was present not only in the transfected neurons but also in neurons neighboring the transfected ones because they could have taken up the BDNF-flag secreted by the transfected ones [45,79]. If so, this would amplify the effect of the transfection recovering the functional re-innervation of the striatum and motor behavior. It should be emphasized that the recovery of the dopamine neurons did not produce dyskinetic movements as occurs in the case of dopaminergic transplants in the striatum [80–83], suggesting that, in the present case, the functional recovery of the striatal innervation was not accompanied by an excessive dopaminergic innervation of the striatum.

## Conclusions

Our findings provide evidence that the continuous and prolonged activation of dopamine D3 receptors associated with the selective non-viral transfection of the BDNF gene to the dopamine neurons of the substantia nigra pars compacta produces a substantial and persistent recovery of motor behavior in an aged rat model of Parkinson´s disease. The motor benefits correlate with an increase in the number of TH+ neurons re-innervating the striatum as well as with the recovery of the spines of striatal medium-sized spiny neurons. A pharmacological effect is unlikely to explain the functional recovery because the improvement in motor behavior persisted at least 2 months after the end of the treatment, suggesting rather a trophic effect. The combined treatment seems to be a promising strategy for recovering the dopamine neurons in this experimental model of Parkinson´s disease, representing initial Parkinson in middle to old age subjects. However, as the traditional toxin model of PD used in this study has focused on the nigrostriatal pathway and the loss of dopamine neurons in this region, it is limited in that it does not reproduce the full pathology and progression seen in PD [84]. This situation creates a need for further work, testing the treatment in different models of the disease to assess its reliability.

## Supporting Information

S1 Video GAIT ANALYSIS IN NORMAL SPEED. (MPG)

S2 Video GAIT ANALYSIS IN SLOW MOTION. (WMV)

S3 Video ROTAROD PERFORMANCE IN NORMAL SPEED. (WMV)

S4 Video ROTAROD PERFORMANCE IN SLOW MOTION. (WMV)

## References

[1] Bernheimer H, Birkmayer W, Hornykiewicz O, Jellinger K, Seitelberger F (1973) Brain dopamine and the syndromes of Parkinson and Huntington. Clinical, morphological and neurochemical correlations. J Neurol Sci 20: 415–455. 4272516 10.1016/0022-510x(73)90175-5

[2] Fearnley JM, Lees AJ (1991) Ageing and Parkinson’s disease: substantia nigra regional selectivity. Brain 114 (Pt 5): 2283–2301. 1933245 10.1093/brain/114.5.2283

[3] Martinez-Fong D, Bannon MJ, Trudeau LE, Gonzalez-Barrios JA, Arango-Rodriguez ML, et al (2012) NTS-Polyplex: a potential nanocarrier for neurotrophic therapy of Parkinson’s disease. Nanomedicine 8: 1052–1069. 10.1016/j.nano.2012.02.009 22406187 PMC3394898

[4] Connor B, Dragunow M (1998) The role of neuronal growth factors in neurodegenerative disorders of the human brain. Brain Res Brain Res Rev 27: 1–39. 9639663 10.1016/s0165-0173(98)00004-6

[5] Lindsay RM (1995) Neuron saving schemes. Nature 373: 289–290. 7830762 10.1038/373289a0

[6] Unsicker K (1994) Growth factors in Parkinson’s disease. Prog Growth Factor Res 5: 73–87. 8199355 10.1016/0955-2235(94)90018-3

[7] Zhou J, Bradford HF, Stern GM (1994) The response of human and rat fetal ventral mesencephalon in culture to the brain-derived neurotrophic factor treatment. Brain Res 656: 147–156. 7804829 10.1016/0006-8993(94)91376-5

[8] Zhou J, Bradford HF, Stern GM (1994) The stimulatory effect of brain-derived neurotrophic factor on dopaminergic phenotype expression of embryonic rat cortical neurons in vitro. Brain Res Dev Brain Res 81: 318–324. 7813052 10.1016/0165-3806(94)90318-2

[9] Im SH, Yu JH, Park ES, Lee JE, Kim HO, et al (2010) Induction of striatal neurogenesis enhances functional recovery in an adult animal model of neonatal hypoxic-ischemic brain injury. Neuroscience 169: 259–268. 10.1016/j.neuroscience.2010.04.038 20610036

[10] Hyman C, Hofer M, Barde YA, Juhasz M, Yancopoulos GD, et al (1991) BDNF is a neurotrophic factor for dopaminergic neurons of the substantia nigra. Nature 350: 230–232. 2005978 10.1038/350230a0

[11] Fritsch B, Reis J, Martinowich K, Schambra HM, Ji Y, et al (2010) Direct current stimulation promotes BDNF-dependent synaptic plasticity: potential implications for motor learning. Neuron 66: 198–204. 10.1016/j.neuron.2010.03.035 20434997 PMC2864780

[12] Goggi J, Pullar IA, Carney SL, Bradford HF (2002) Modulation of neurotransmitter release induced by brain-derived neurotrophic factor in rat brain striatal slices in vitro. Brain Res 941: 34–42. 12031545 10.1016/s0006-8993(02)02505-2

[13] Blochl A, Sirrenberg C (1996) Neurotrophins stimulate the release of dopamine from rat mesencephalic neurons via Trk and p75Lntr receptors. J Biol Chem 271: 21100–21107. 8702878 10.1074/jbc.271.35.21100

[14] Shen RY, Altar CA, Chiodo LA (1994) Brain-derived neurotrophic factor increases the electrical activity of pars compacta dopamine neurons in vivo. Proc Natl Acad Sci U S A 91: 8920–8924. 8090745 10.1073/pnas.91.19.8920 PMC44718

[15] Yurek DM, Lu W, Hipkens S, Wiegand SJ (1996) BDNF enhances the functional reinnervation of the striatum by grafted fetal dopamine neurons. Exp Neurol 137: 105–118. 8566202 10.1006/exnr.1996.0011

[16] Zhou J, Bradford HF, Stern GM (1997) Influence of BDNF on the expression of the dopaminergic phenotype of tissue used for brain transplants. Brain Res Dev Brain Res 100: 43–51. 9174245 10.1016/s0165-3806(97)00019-9

[17] Guillin O, Diaz J, Carroll P, Griffon N, Schwartz JC, et al (2001) BDNF controls dopamine D3 receptor expression and triggers behavioural sensitization. Nature 411: 86–89. 11333982 10.1038/35075076

[18] Sokoloff P, Guillin O, Diaz J, Carroll P, Griffon N (2002) Brain-derived neurotrophic factor controls dopamine D3 receptor expression: implications for neurodevelopmental psychiatric disorders. Neurotox Res 4: 671–678. 12709305 10.1080/1029842021000045499

[19] Howells DW, Porritt MJ, Wong JY, Batchelor PE, Kalnins R, et al (2000) Reduced BDNF mRNA expression in the Parkinson’s disease substantia nigra. Exp Neurol 166: 127–135. 11031089 10.1006/exnr.2000.7483

[20] Porritt M, Stanic D, Finkelstein D, Batchelor P, Lockhart S, et al (2005) Dopaminergic innervation of the human striatum in Parkinson’s disease. Mov Disord 20: 810–818. 15726582 10.1002/mds.20399

[21] Mogi M, Togari A, Kondo T, Mizuno Y, Komure O, et al (1999) Brain-derived growth factor and nerve growth factor concentrations are decreased in the substantia nigra in Parkinson’s disease. Neurosci Lett 270: 45–48. 10454142 10.1016/s0304-3940(99)00463-2

[22] Venero JL, Beck KD, Hefti F (1994) 6-Hydroxydopamine lesions reduce BDNF mRNA levels in adult rat brain substantia nigra. Neuroreport 5: 429–432. 8003668 10.1097/00001756-199401120-00014

[23] Van Kampen JM, Hagg T, Robertson HA (2004) Induction of neurogenesis in the adult rat subventricular zone and neostriatum following dopamine D3 receptor stimulation. Eur J Neurosci 19: 2377–2387. 15128392 10.1111/j.0953-816X.2004.03342.x

[24] Kim Y, Wang WZ, Comte I, Pastrana E, Tran PB, et al (2010) Dopamine stimulation of postnatal murine subventricular zone neurogenesis via the D3 receptor. J Neurochem 114: 750–760. 10.1111/j.1471-4159.2010.06799.x 20477937 PMC2913229

[25] Pilon C, Levesque D, Dimitriadou V, Griffon N, Martres MP, et al (1994) Functional coupling of the human dopamine D3 receptor in a transfected NG 108–15 neuroblastoma-glioma hybrid cell line. Eur J Pharmacol 268: 129–139. 7957635 10.1016/0922-4106(94)90182-1

[26] Chio CL, Lajiness ME, Huff RM (1994) Activation of heterologously expressed D3 dopamine receptors: comparison with D2 dopamine receptors. Mol Pharmacol 45: 51–60. 8302280

[27] Griffon N, Pilon C, Sautel F, Schwartz JC, Sokoloff P (1997) Two intracellular signaling pathways for the dopamine D3 receptor: opposite and synergistic interactions with cyclic AMP. J Neurochem 68: 1–9. 8978703 10.1046/j.1471-4159.1997.68010001.x

[28] Collo G, Zanetti S, Missale C, Spano P (2008) Dopamine D3 receptor-preferring agonists increase dendrite arborization of mesencephalic dopaminergic neurons via extracellular signal-regulated kinase phosphorylation. Eur J Neurosci 28: 1231–1240. 10.1111/j.1460-9568.2008.06423.x 18973551

[29] Levesque D, Martres MP, Diaz J, Griffon N, Lammers CH, et al (1995) A paradoxical regulation of the dopamine D3 receptor expression suggests the involvement of an anterograde factor from dopamine neurons. Proc Natl Acad Sci U S A 92: 1719–1723. 7878047 10.1073/pnas.92.5.1719 PMC42591

[30] Ryoo HL, Pierrotti D, Joyce JN (1998) Dopamine D3 receptor is decreased and D2 receptor is elevated in the striatum of Parkinson’s disease. Mov Disord 13: 788–797. 9756147 10.1002/mds.870130506

[31] Van Kampen JM, Eckman CB (2006) Dopamine D3 receptor agonist delivery to a model of Parkinson’s disease restores the nigrostriatal pathway and improves locomotor behavior. J Neurosci 26: 7272–7280. 16822985 10.1523/JNEUROSCI.0837-06.2006 PMC6673939

[32] Du F, Li R, Huang Y, Li X, Le W (2005) Dopamine D3 receptor-preferring agonists induce neurotrophic effects on mesencephalic dopamine neurons. Eur J Neurosci 22: 2422–2430. 16307585 10.1111/j.1460-9568.2005.04438.x

[33] Diaz J, Ridray S, Mignon V, Griffon N, Schwartz JC, et al (1997) Selective expression of dopamine D3 receptor mRNA in proliferative zones during embryonic development of the rat brain. J Neurosci 17: 4282–4292. 9151745 10.1523/JNEUROSCI.17-11-04282.1997 PMC6573556

[34] Razgado LF, Sierra A, Anaya-Martinez V, Jimenez I, Martinez-Fong D, et al. (2013) Activation of dopamine D3 receptors combined with the BDNF gene transfection to the dopamine cells of the pars compacta recovers the striatal innervation and motor behavior in the rat model of Parkinson´s disease Soc Neurosci Abstr 13802/G34

[35] Rozas G, Labandeira Garcia JL (1997) Drug-free evaluation of rat models of parkinsonism and nigral grafts using a new automated rotarod test. Brain Res 749: 188–199. 9138718 10.1016/S0006-8993(96)01162-6

[36] Kirik D, Rosenblad C, Bjorklund A (1998) Characterization of behavioral and neurodegenerative changes following partial lesions of the nigrostriatal dopamine system induced by intrastriatal 6-hydroxydopamine in the rat. Exp Neurol 152: 259–277. 9710526 10.1006/exnr.1998.6848

[37] Voorn P, Vanderschuren LJ, Groenewegen HJ, Robbins TW, Pennartz CM (2004) Putting a spin on the dorsal-ventral divide of the striatum. Trends Neurosci 27: 468–474. 15271494 10.1016/j.tins.2004.06.006

[38] Graybiel AM (2005) The basal ganglia: learning new tricks and loving it. Curr Opin Neurobiol 15: 638–644. 16271465 10.1016/j.conb.2005.10.006

[39] Paxinos G, Watson C (1998) The rat brain: academic press 117 p.

[40] Casarrubea M, Sorbera F, Crescimanno G (2006) Effects of 7-OH-DPAT and U 99194 on the behavioral response to hot plate test, in rats. Physiol Behav 89: 552–562. 16919688 10.1016/j.physbeh.2006.07.014

[41] Gonzalez-Barrios JA, Lindahl M, Bannon MJ, Anaya-Martinez V, Flores G, et al (2006) Neurotensin polyplex as an efficient carrier for delivering the human GDNF gene into nigral dopamine neurons of hemiparkinsonian rats. Mol Ther 14: 857–865. 17015039 10.1016/j.ymthe.2006.09.001

[42] Hernandez-Baltazar D, Martinez-Fong D, Trudeau LE (2012) Optimizing NTS-polyplex as a tool for gene transfer to cultured dopamine neurons. PLoS One 7: e51341 10.1371/journal.pone.0051341 23300540 PMC3530538

[43] Alvarez-Maya I, Navarro-Quiroga I, Meraz-Rios MA, Aceves J, Martinez-Fong D (2001) In vivo gene transfer to dopamine neurons of rat substantia nigra via the high-affinity neurotensin receptor. Mol Med 7: 186–192. 11471555 PMC1950024

[44] Navarro-Quiroga I, Antonio Gonzalez-Barrios J, Barron-Moreno F, Gonzalez-Bernal V, Martinez-Arguelles DB, et al (2002) Improved neurotensin-vector-mediated gene transfer by the coupling of hemagglutinin HA2 fusogenic peptide and Vp1 SV40 nuclear localization signal. Brain Res Mol Brain Res 105: 86–97. 12399111 10.1016/s0169-328x(02)00396-0

[45] Arango-Rodriguez ML, Navarro-Quiroga I, Gonzalez-Barrios JA, Martinez-Arguelles DB, Bannon MJ, et al (2006) Biophysical characteristics of neurotensin polyplex for in vitro and in vivo gene transfection. Biochim Biophys Acta 1760: 1009–1020. 16730907 10.1016/j.bbagen.2006.02.021

[46] Sacchetti P, Brownschidle LA, Granneman JG, Bannon MJ (1999) Characterization of the 5′-flanking region of the human dopamine transporter gene. Brain Res Mol Brain Res 74: 167–174. 10640687 10.1016/s0169-328x(99)00275-2

[47] Avila-Costa MR, Colin-Barenque L, Fortoul TI, Machado-Salas P, Espinosa-Villanueva J, et al (1999) Memory deterioration in an oxidative stress model and its correlation with cytological changes on rat hippocampus CA1. Neurosci Lett 270: 107–109. 10462109 10.1016/s0304-3940(99)00458-9

[48] Avila-Costa MR, Montiel Flores E, Colin-Barenque L, Ordonez JL, Gutierrez AL, et al (2004) Nigrostriatal modifications after vanadium inhalation: an immunocytochemical and cytological approach. Neurochem Res 29: 1365–1369. 15202766 10.1023/b:nere.0000026398.86113.7d

[49] Wang XH, Lu G, Hu X, Tsang KS, Kwong WH, et al (2012) Quantitative assessment of gait and neurochemical correlation in a classical murine model of Parkinson’s disease. BMC Neurosci 13: 142 10.1186/1471-2202-13-142 23151254 PMC3507899

[50] Filipe VM, Pereira JE, Costa LM, Mauricio AC, Couto PA, et al (2006) Effect of skin movement on the analysis of hindlimb kinematics during treadmill locomotion in rats. J Neurosci Methods 153: 55–61. 16337686 10.1016/j.jneumeth.2005.10.006

[51] Durieux PF, Schiffmann SN, de Kerchove d’Exaerde A (2012) Differential regulation of motor control and response to dopaminergic drugs by D1R and D2R neurons in distinct dorsal striatum subregions. EMBO J 31: 640–653. 10.1038/emboj.2011.400 22068054 PMC3273396

[52] Brown LL, Sharp FR (1995) Metabolic mapping of rat striatum: somatotopic organization of sensorimotor activity. Brain Res 686: 207–222. 7583286 10.1016/0006-8993(95)00457-2

[53] Brown LL (1992) Somatotopic organization in rat striatum: evidence for a combinational map. Proc Natl Acad Sci U S A 89: 7403–7407. 1502150 10.1073/pnas.89.16.7403 PMC49718

[54] Rozas G, Lopez-Martin E, Guerra MJ, Labandeira-Garcia JL (1998) The overall rod performance test in the MPTP-treated-mouse model of Parkinsonism. J Neurosci Methods 83: 165–175. 9765130 10.1016/s0165-0270(98)00078-8

[55] Metz GA, Tse A, Ballermann M, Smith LK, Fouad K (2005) The unilateral 6-OHDA rat model of Parkinson’s disease revisited: an electromyographic and behavioural analysis. Eur J Neurosci 22: 735–744. 16101755 10.1111/j.1460-9568.2005.04238.x

[56] Lee HY, Hsieh TH, Liang JI, Yeh ML, Chen JJ (2012) Quantitative video-based gait pattern analysis for hemiparkinsonian rats. Med Biol Eng Comput 50: 937–946. 10.1007/s11517-012-0933-5 22707230

[57] Walsh RN, Cummins RA (1976) The Open-Field Test: a critical review. Psychol Bull 83: 482–504. 17582919

[58] Carola V, D’Olimpio F, Brunamonti E, Mangia F, Renzi P (2002) Evaluation of the elevated plus-maze and open-field tests for the assessment of anxiety-related behaviour in inbred mice. Behav Brain Res 134: 49–57. 12191791 10.1016/s0166-4328(01)00452-1

[59] Prut L, Belzung C (2003) The open field as a paradigm to measure the effects of drugs on anxiety-like behaviors: a review. Eur J Pharmacol 463: 3–33. 12600700 10.1016/s0014-2999(03)01272-x

[60] Cenci MA, Campbell K, Wictorin K, Bjorklund A (1992) Striatal c-fos Induction by Cocaine or Apomorphine Occurs Preferentially in Output Neurons Projecting to the Substantia Nigra in the Rat. Eur J Neurosci 4: 376–380. 12106364 10.1111/j.1460-9568.1992.tb00885.x

[61] Wictorin K, Simerly RB, Isacson O, Swanson LW, Bjorklund A (1989) Connectivity of striatal grafts implanted into the ibotenic acid-lesioned striatum—III. Efferent projecting graft neurons and their relation to host afferents within the grafts. Neuroscience 30: 313–330. 2747920 10.1016/0306-4522(89)90256-x

[62] Lindner MD, Cain CK, Plone MA, Frydel BR, Blaney TJ, et al (1999) Incomplete nigrostriatal dopaminergic cell loss and partial reductions in striatal dopamine produce akinesia, rigidity, tremor and cognitive deficits in middle-aged rats. Behav Brain Res 102: 1–16. 10403011 10.1016/s0166-4328(98)00160-0

[63] Dickerson JW, Hemmerle AM, Numan S, Lundgren KH, Seroogy KB (2009) Decreased expression of ErbB4 and tyrosine hydroxylase mRNA and protein in the ventral midbrain of aged rats. Neuroscience 163: 482–489. 10.1016/j.neuroscience.2009.06.008 19505538 PMC2755587

[64] Singh S, Ahmad R, Mathur D, Sagar RK, Krishana B (2006) Neuroprotective effect of BDNF in young and aged 6-OHDA treated rat model of Parkinson disease. Indian J Exp Biol 44: 699–704. 16999024

[65] Connor B, Kozlowski DA, Schallert T, Tillerson JL, Davidson BL, et al (1999) Differential effects of glial cell line-derived neurotrophic factor (GDNF) in the striatum and substantia nigra of the aged Parkinsonian rat. Gene Ther 6: 1936–1951. 10637445 10.1038/sj.gt.3301033

[66] Polinski NK, Gombash SE, Manfredsson FP, Lipton JW, Kemp CJ, et al (2014) Recombinant adenoassociated virus 2/5-mediated gene transfer is reduced in the aged rat midbrain. Neurobiol Aging. 10.1016/j.neurobiolaging.2014.11.019 25457558 PMC4315740

[67] Coronas V, Bantubungi K, Fombonne J, Krantic S, Schiffmann SN, et al (2004) Dopamine D3 receptor stimulation promotes the proliferation of cells derived from the post-natal subventricular zone. J Neurochem 91: 1292–1301. 15584906 10.1111/j.1471-4159.2004.02823.x

[68] Van Kampen JM, Robertson HA (2005) A possible role for dopamine D3 receptor stimulation in the induction of neurogenesis in the adult rat substantia nigra. Neuroscience 136: 381–386. 16216425 10.1016/j.neuroscience.2005.07.054

[69] Solis O, Limon DI, Flores-Hernandez J, Flores G (2007) Alterations in dendritic morphology of the prefrontal cortical and striatum neurons in the unilateral 6-OHDA-rat model of Parkinson’s disease. Synapse 61: 450–458. 17372982 10.1002/syn.20381

[70] Zaja-Milatovic S, Milatovic D, Schantz AM, Zhang J, Montine KS, et al (2005) Dendritic degeneration in neostriatal medium spiny neurons in Parkinson disease. Neurology 64: 545–547. 15699393 10.1212/01.WNL.0000150591.33787.A4

[71] Stephens B, Mueller AJ, Shering AF, Hood SH, Taggart P, et al (2005) Evidence of a breakdown of corticostriatal connections in Parkinson’s disease. Neuroscience 132: 741–754. 15837135 10.1016/j.neuroscience.2005.01.007

[72] Ingham CA, Hood SH, Taggart P, Arbuthnott GW (1998) Plasticity of synapses in the rat neostriatum after unilateral lesion of the nigrostriatal dopaminergic pathway. J Neurosci 18: 4732–4743. 9614247 10.1523/JNEUROSCI.18-12-04732.1998 PMC6792704

[73] Soderstrom KE, O’Malley JA, Levine ND, Sortwell CE, Collier TJ, et al (2010) Impact of dendritic spine preservation in medium spiny neurons on dopamine graft efficacy and the expression of dyskinesias in parkinsonian rats. Eur J Neurosci 31: 478–490. 10.1111/j.1460-9568.2010.07077.x 20105237 PMC2940228

[74] Double KL, Crocker AD (1995) Dopamine receptors in the substantia nigra are involved in the regulation of muscle tone. Proc Natl Acad Sci U S A 92: 1669–1673. 7878037 10.1073/pnas.92.5.1669 PMC42581

[75] Takakusaki K, Habaguchi T, Ohtinata-Sugimoto J, Saitoh K, Sakamoto T (2003) Basal ganglia efferents to the brainstem centers controlling postural muscle tone and locomotion: a new concept for understanding motor disorders in basal ganglia dysfunction. Neuroscience 119: 293–308. 12763089 10.1016/s0306-4522(03)00095-2

[76] Takakusaki K, Tomita N, Yano M (2008) Substrates for normal gait and pathophysiology of gait disturbances with respect to the basal ganglia dysfunction. J Neurol 255 Suppl 4: 19–29. 10.1007/s00415-008-4004-7 18821082

[77] Glajch KE, Fleming SM, Surmeier DJ, Osten P (2012) Sensorimotor assessment of the unilateral 6-hydroxydopamine mouse model of Parkinson’s disease. Behav Brain Res 230: 309–316. 10.1016/j.bbr.2011.12.007 22178078 PMC3324279

[78] Eskow Jaunarajs KL, Dupre KB, Ostock CY, Button T, Deak T, et al (2010) Behavioral and neurochemical effects of chronic L-DOPA treatment on nonmotor sequelae in the hemiparkinsonian rat. Behav Pharmacol 21: 627–637. 10.1097/FBP.0b013e32833e7e80 20838211 PMC2953710

[79] Zheng J, Shen WH, Lu TJ, Zhou Y, Chen Q, et al (2008) Clathrin-dependent endocytosis is required for TrkB-dependent Akt-mediated neuronal protection and dendritic growth. J Biol Chem 283: 13280–13288. 10.1074/jbc.M709930200 18353779

[80] Shin E, Lisci C, Tronci E, Fidalgo C, Stancampiano R, et al (2014) The anti-dyskinetic effect of dopamine receptor blockade is enhanced in parkinsonian rats following dopamine neuron transplantation. Neurobiol Dis 62: 233–240. 10.1016/j.nbd.2013.09.021 24135006

[81] Politis M, Oertel WH, Wu K, Quinn NP, Pogarell O, et al (2011) Graft-induced dyskinesias in Parkinson’s disease: High striatal serotonin/dopamine transporter ratio. Mov Disord 26: 1997–2003. 10.1002/mds.23743 21611977

[82] Vinuela A, Hallett PJ, Reske-Nielsen C, Patterson M, Sotnikova TD, et al (2008) Implanted reuptake-deficient or wild-type dopaminergic neurons improve ON L-dopa dyskinesias without OFF-dyskinesias in a rat model of Parkinson’s disease. Brain 131: 3361–3379. 10.1093/brain/awn192 18988638 PMC2639209

[83] Ma Y, Peng S, Dhawan V, Eidelberg D (2011) Dopamine cell transplantation in Parkinson’s disease: challenge and perspective. Br Med Bull 100: 173–189. 10.1093/bmb/ldr040 21875864 PMC3276236

[84] Fleming SM, Fernagut PO, Chesselet MF (2005) Genetic mouse models of parkinsonism: strengths and limitations. NeuroRx 2: 495–503. 16389313 10.1602/neurorx.2.3.495 PMC1144493

